# Co-Creating a Health Equity Journey: Participatory Action Research With Older Black Adults

**DOI:** 10.1093/geroni/igaf122.1262

**Published:** 2025-12-31

**Authors:** Laura Heinemann, LaShaune Johnson

**Affiliations:** Creighton University, Omaha, Nebraska, United States; Tilman J. Fertitta Family College of Medicine, University of Houston, Houston, Texas, United States

## Abstract

We present the story of an interdisciplinary participatory-action research project for addressing health-inequities among older Black adults who had experienced hospitalization. Melding social science, humanities, arts-based approaches, and local epistemologies, we presupposed project outcomes required guidance and partnership from older adults with direct knowledge and lived experiences of the consequences of health inequities. Working within a historically Black Midwestern urban community impacted by redlining and disinvestment, our multigenerational team included a public health-trained faith leader and gerontological social worker, alongside faculty and students from social science, studio art, public health, and medicine. Honoring the African tradition of griots (travelling storytellers), narrative interviews about hospitalization segued into weekly conversation hours and arts-based activities where community residents imparted insights from their broader life journeys. Seeking to ensure non-harmful, culturally responsive representation of study findings, we applied Peak and Anderson’s (2018) Journey Scroll technique by offering spoken and graphic-novel-style visual retelling of participants’ collective stories. During community data “share-back” events, community residents were invited to see, discuss, interpret, and potentially challenge or reframe study findings. Together with local organizations, residents, and leaders long-committed to Black community wellbeing, the project co-created emerging methodologies for inquiry and action. Attendees for this paper will be able to 1) recognize W. Andrew Achenbaum’s legacy of connecting humanities and gerontology through community-participatory collaborations; 2) appreciate lessons and new possibilities for weaving together social science and humanistic approaches to support older Black adults’ well-being; 3) leverage incorporation of intergenerational and multidisciplinary research teams toward addressing health-inequities.

